# Brain Tax: Estimating the Population-Level Impact of Environmental Chemicals on IQ Scores

**DOI:** 10.1289/ehp.120-a165b

**Published:** 2012-04-01

**Authors:** Wendee Holtcamp

**Affiliations:** Houston-based science writer Wendee Holtcamp has written for *Miller-McCune*, *Scientific American*, and other magazines.

Many studies report the impact of various environmental chemicals on children’s neurological development as “clinically insignificant.” But this designation, which describes the individual child, does not always reflect the broader implications of exposure for the overall population—i.e., that a mild but frequent impact could add up to a substantial population-level burden. A new analysis establishes priorities for reducing adverse neurodevelopmental impacts for U.S. children aged 5 years and younger [*EHP* 120(4):501–507; Bellinger].

The author assessed the population impact of various risk factors, including exposure to environmental chemicals, on Full-Scale IQ (FSIQ) scores. FSIQ values are a useful measure for such an assessment because past studies have shown that these scores correspond to work productivity and associated lifetime earnings. FSIQ declines therefore can be used to estimate economic consequences for various risk factors.

The data necessary for such an analysis, including information on both prevalence of exposure and effects, were available for only 3 environmental chemicals: lead, methylmercury, and organophosphate pesticides. The loss in FSIQ points was also calculated for a variety of medical conditions, neurodevelopmental disorders, traumatic brain injury, failure to thrive as a result of neglect and abuse, and iron deficiency. Loss of IQ points attributable to each risk factor was estimated based on meta-analyses or pooled analyses of existing data, then generalized to the 25.5 million children aged 0–5 years estimated to live in the United States, based on the prevalence of exposure to each risk factor.

Preterm birth resulted in the largest population health burden, with estimated losses of more than 34 million IQ points populationwide, followed by lead exposure at 22.9 million points lost. Organophosphate pesticides and attention deficit/hyperactivity disorder both resulted in estimated losses of more than 16 million points, iron deficiency in 9.4 million points, pediatric bipolar disorder in 8 million points, autism spectrum disorders in 7 million points, brain injury in 5.8 million points, and failure to thrive in 5.3 million points. The remaining risk factors resulted in estimated cumulative IQ losses of fewer than 1 million points each. Interactions between factors certainly exist, although the current study was not able to assess these.

A surprising finding was that a large fraction of the total estimated IQ loss associated with lead was contributed by children in the lower reaches of the blood lead distribution—that’s because so many children have low levels of exposure. But the author estimates that reductions in U.S. lead exposure nevertheless “saved” more than 100 million IQ points between the 1976–1980 and 2005-2006 iterations of the National Health and Nutrition Examination Survey. This suggests that similar reductions in nations where lead exposures remain high could result in dramatic improvements in neurodevelopment on a population level.

